# Possible Effect of Governmental Restrictions of Benzodiazepine Prescriptions on Suicide Attempts by Overdose of Prescribed Psychotropic Drugs in Japan

**DOI:** 10.1002/npr2.70068

**Published:** 2025-12-08

**Authors:** Akihisa Akahane, Kenichi Matsumura, Yukari Mashiko, Rie Kanai, Hiroshi Kunugi

**Affiliations:** ^1^ Department of Psychiatry Teikyo University School of Medicine Tokyo Japan

**Keywords:** benzodiazepine, medical policy, overdose, suicide

## Abstract

**Purpose:**

Suicide attempt by overdose medication of benzodiazepine is common. In Japan, governmental restrictions on benzodiazepine multidrug use started as of 2012, resulting in a decrease in benzodiazepine prescriptions and multidrug use. This study aimed to examine whether suicide attempts due to overdose medication of psychotropic drugs decreased after this regulation.

**Methods:**

We retrospectively obtained information from clinical records for 4183 and 4140 patients admitted to the intensive care unit of the Advanced Emergency Medical Center at Teikyo University Hospital, Tokyo, during the 2‐year period from April 2013 to March 2015 (immediately after the introduction of the regulation: Period 1) and from April 2018 to March 2020 (after the enhancement of the regulations: Period 2), respectively. The proportion of patients who attempted suicide, that of overmedication with prescribed psychotropic drugs, and their clinical characteristics were compared between the two periods.

**Results:**

The proportion of patients with suicide attempts by psychotropic overdose medication in Period 1 was 4.1%, whereas it decreased significantly to 2.8% in Period 2 (*p* = 0.004). The mean (standard deviation) diazepam‐equivalent daily prescription for those overdosing was 32.0 (33.3) mg in Period 1, compared to 25.6 (30.0) mg in Period 2, a significant decrease (*p* = 0.01). The mean number of concomitant benzodiazepines also decreased significantly from 2.8 (1.4) to 2.0 (1.0) (*p* = 0.0002).

**Conclusions:**

Our results suggest that the governmental regulation to control multidrug use of benzodiazepine prescriptions in Japan reduced the number and doses of benzodiazepines prescribed and decreased suicide attempts by overdose medication of prescribed psychotropic drugs.

## Abstract 2

This study aimed to examine whether suicide attempts due to overdose medication of psychotropic drugs decreased after governmental regulations (as of 2012) on benzodiazepine multidrug use. We compared patients with attempted suicide who were admitted to the intensive care unit immediately after the introduction of the governmental regulation (April 2013 to March 2015) and those patients admitted after the enhancement of the regulations (April 2018 to March 2020). Our results suggest that the governmental regulations to control multidrug use of benzodiazepine prescriptions reduced the number and doses of benzodiazepines prescribed and decreased suicide attempts by overdose medication of prescribed psychotropic drugs.

## Graphical abstract

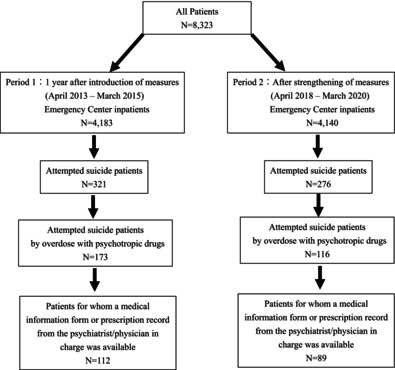

## 1 Introduction

The percentage of suicide attempts among all emergency room visits in Japan is reported to be 4.7% [1], which is about 10 times higher than that in the United States (0.4%). The most frequent means of suicide is overdose in Japan (52%), in the United States (68%), and the United Kingdom (78%). In recent years, opioids have been the most common drug used in Europe and the United States, while benzodiazepines account for the majority of overdoses in Japan [2].

It is therefore important to examine the pharmacotherapy received by patients who attempt suicide by overdose medication with psychotropic drugs for the treatment and prevention of suicide. We previously conducted a retrospective study on prescriptions at the time of suicide attempt of patients who used overdose medication with psychotropic drugs. The studies had been conducted using medical information data from October 2003 to September 2004 [3] and from August 2009 to July 2010 [4, 5] and noted several characteristics of prescriptions in patients who attempted suicide by overdose with psychotropic medications. In such patients, benzodiazepine prescriptions were well above the maximum daily diazepam equivalent of 15 mg, and the rate of patients who received any antidepressant prescription was quite low.

In Japan, the Basic Law on Suicide Prevention was enacted in 2006 in response to an increase in the number of suicides. In 2010, the Ministry of Health, Labor and Welfare (MHLW) issued an alert and warning against excessive prescriptions of psychotropic drugs, followed by issuing restrictions in 2012 on health insurance reimbursement aimed at discouraging multiple prescriptions of psychotropic drugs and their prolonged use. The reimbursement to physicians was reduced when more than three types of anti‐anxiety drugs or three types of sleeping pills were prescribed at one time. Since then, this prescribing control measure has been strengthened three times until 2018; that is, expanding the scope of subtraction to non‐psychiatric clinics, increasing the subtraction rate, and eventually introducing the subtraction for benzodiazepines administered continuously for more than 1 year. As a result, according to an analysis using the national database, the rate of benzodiazepine multidrug use (3 or more drugs) decreased significantly since 2015 [6]. The rate of prescribing any anti‐anxiety drugs peaked in 2007 and has continued to decline, and the rate of sleeping pills peaked in 2012 and has been slowly declining [6]. In other countries, however, no clear reduction in prescription was observed after regulations on benzodiazepines in the United States (where the measures were introduced in 2006) [7] or in France (introduced in 2012) [8], while a reduction in prescriptions did occur in the Netherlands (introduced in 2009) [9].

It is possible that the regulation on benzodiazepine prescriptions in Japan had a reducing effect on suicide attempts due to overmedication. However, the association between benzodiazepine prescription restrictions and suicide risk has not yet been fully investigated. In this study, we examined the temporal association between the governmental regulation to control benzodiazepine prescriptions and suicide attempts by overdose medication of prescribed drugs.

## 2 Methods

**FIGURE 1:**
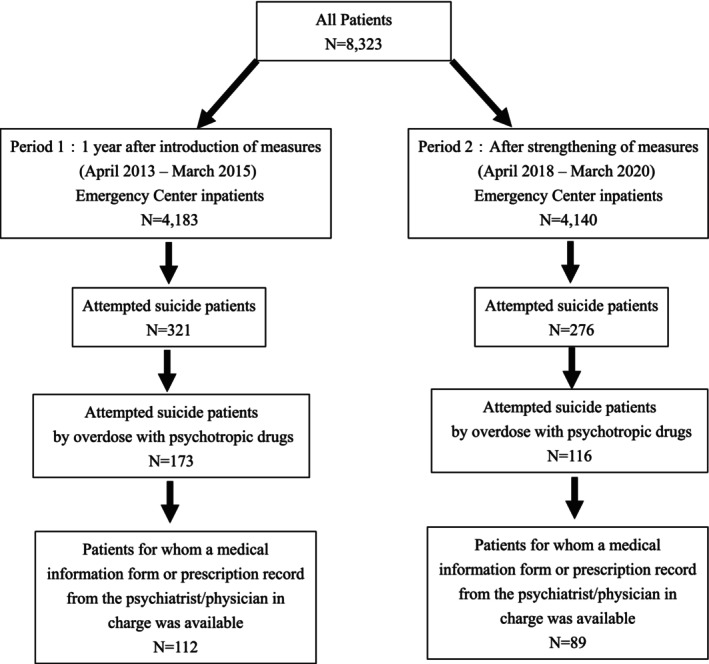
Flowchart of selecting target patients.

A flowchart of the selection of study patients is shown in Figure 1. Teikyo University Hospital is an emergency medical institution located in Tokyo with 12 000 emergency visits per year, including walk‐in patients to the ER (Emergency Room). There have been approximately 2000 admissions per year to the Intensive Care Unit (ICU) of the Advanced Critical Care Center. Our liaison psychiatry team started to construct systematic clinical data collection in ICU for research as of 2013. Therefore, we set target patients as those admitted to the ICU during the 2‐year period from April 2013 to March 2015 (immediately after the introduction of prescription control measures for benzodiazepines: Period 1) and those from April 2018 to March 2020 (after the measures were strengthened: Period 2). During Periods 1 and 2, 4183 and 4140 patients were admitted to the ICU, respectively.

We compared the proportion of patients who attempted suicide and that of patients who attempted suicide by overdose medication with prescribed psychotropic drugs between Periods 1 and 2. Attempted suicide was defined as an act based on a clear intent to commit suicide (anticipated lethality) of one's own volition.

Patients who attempted suicide by overmedication with prescribed psychotropic drugs were retrospectively surveyed for gender, age, psychiatric diagnosis, and history of suicide attempts based on medical records, and compared between Period 1 (173 patients) and Period 2 (116 patients). Information on prescriptions by their psychiatrists/physicians in charge was available for 112 patients in Period 1 and 89 patients in Period 2. We obtained information on benzodiazepine prescriptions, including drug name, dose, and number of concomitant medications, and then compared them between the two periods. Doses were converted to diazepam based on equivalence conversion [10]. The psychiatric diagnosis was determined based on the International Classification of Diseases (ICD‐10) of the World Health Organization (WHO) [11], by discussion among all psychiatrists in our psychiatric liaison team, based on all available medical information including the medical information form (only one main diagnosis for each patient was used for the analysis in this study).

SPSS Statistics V28.0 was used for statistical analysis. The χ^2^ test or Fisher's exact test was used to compare proportions among categories, and the Mann–Whitney *U* test was used to compare scale/order variables, with a significance level of *p* = 0.05 (two‐tailed). We made five key comparisons between the two periods (proportion of ICU patients with attempted suicide, proportion of ICU patients with overdoses, proportion of overdoses among all suicide attempters, mean daily diazepam equivalent dose, and mean number of concomitant medications). To account for multiple testing, we applied the Bonferroni correction by multiplying each uncorrected *p*‐value by 5. This study was conducted with the approval of the Teikyo University School of Medicine Research Ethics Committee (Teirin No. 21‐716). Since this study is a retrospective observational study and used only information from medical records, individual patients did not confer informed consent. However, to guarantee the right to refuse the use of medical information for research purposes, information about the study was made public on the Teikyo University Ethics Committee's website (https://www.teikyo‐u.ac.jp/application/files/1816/3973/3858/public_projects_202112_06.pdf).

## 3 Results

Comparisons in proportions of patients with attempted suicide between Periods 1 and 2 are shown in Table 1. The proportions of patients with attempted suicide among those who were admitted to the ICU in Periods 1 and 2 were 7.7% (321/4183) and 6.7% (276/4140), respectively, showing no significant difference (χ^2^ = 3.17, df = 1, *p* = 0.07, corrected *p* = 0.35). Of these, the proportion of patients who attempted suicide by overdose medication with prescribed psychotropic drugs was 4.1% (173/4183) in Period 1% and 2.8% (116/4140) in Period 2, showing a significant decrease (χ^2^ = 11.04, df = 1, *p* = 0.001, φ = 0.04, corrected *p* = 0.005). There was also a significant decrease in the proportion of patients who attempted suicide by overdose medication of prescribed psychotropic drugs among total patients with attempted suicide (Period 1: 53.9% [173/321 patients]; Period 2: 42.0% [116/276 patients]) (χ^2^ = 8.37, df = 1, *p* = 0.004, φ = 0.12, corrected *p* = 0.02).

**TABLE 1:** Comparisons in proportions of patients with attempted suicide between Periods 1 and 2.

Attempted suicide (AS)	Period 1 (2013–2015)	Period 2 (2018–2020)	*p* (corrected)
Proportion of AS in ICU patients	7.7% (321/4183)	6.7% (276/4140)	*P* = 0.07 (*p* = 0.35)
Proportion of OD in ICU patients	4.1% (173/4183)	2.8% (116/4140)	*P* = 0.001 (*P* = 0.005)
Proportion of OD in total AS	53.9% (173/321)	42.0% (116/276)	*P* = 0.004 (*P* = 0.02)

Abbreviations: ICU, intensive care unit; OD, overdose medication by prescribed psychotropic drugs.

Clinical characteristics of patients who attempted suicide by overdose medication with prescribed psychotropic drugs are shown in Table 2. There was no significant difference in gender, age, or history of attempted suicide between the 2 Periods. In terms of benzodiazepine prescription status among patients for whom prescription information was available, mean daily diazepam equivalent dose (± standard deviation) was significantly lower in Period 2 (25.6 ± 30.0 mg) than in Period 1 (32.0 ± 33.3 mg) (*U* = 3923.0, *Z* = −2.59, *p* = 0.01, *γ* = 0.18, corrected *p* = 0.048) (see Table 3). The mean number of concomitant medications was also significantly lower in Period 2 (2.0 ± 1.0), compared with Period 1 (2.8 ± 1.4) (*U* = 3490.5, *Z* = −3.78, *p* = 0.0002, *γ* = 0.27, corrected *p* = 0.001).

**TABLE 2:** Clinical characteristics of attempted suicide patients by overdose medication with psychotropic drugs.

	Period 1 (2013–2015)		Period 2 (2018–2020)		*p*
	*N* = 173		*N* = 116		
	*n*	%	*n*	%	
Gender					
Female	126	72.8	94	81.0	*p* = 0.12^a^
Age					
9 ~ 19 years	5	2.9	8	6.9	
20 ~ 29 years	52	30.1	31	26.7	
30 ~ 39 years	53	30.6	29	25.0	
40 ~ 49 years	39	22.5	23	19.8	
50 ~ 59 years	13	7.5	12	10.3	
60 ~ 69 years	6	3.5	7	6.0	
≧ 70 years	5	2.9	6	5.2	
Mean age, years (SD)	37.0 (13.0)		38.7 (16.0)		*p* = 0.35^b^
Diagnosis (ICD‐10)					
Alzheimer disease (F00)	2	1.2	0	0	
Organic mental disorder (F06)	0	0	2	1.7	
Alcoholism (F10)	2	1.2	1	0.9	
Benzodiazepine dependence (F13)	3	1.7	0	0	
Post‐methamphetamine (F15)	1	0.6	0	0	
Schizophrenia (F20)	31	17.9	15	12.9	
Delusional disorder (F22)	1	0.6	0	0	
Schizoaffective disorder (F25)	1	0.6	0	0	
Bipolar disorder (F31)	32	18.5	12	10.3	
Depression (F32)	34	19.7	25	21.6	
Dysthymia (F34)	0	0	2	1.7	
Social anxiety disorder (F40)	0	0	1	0.9	
Anxiety disorder (F41)	10	5.8	4	3.4	
Adjustment disorder (F43)	27	15.6	28	24.1	
PTSD (F43)	2	1.2	1	0.9	
Dissociative disorder (F44)	1	0.6	0	0	
Eating disorder (F50)	4	2.3	4	3.4	
Personality disorder (F60)	16	9.2	11	9.5	
Gender identity disorder (F64)	0	0	1	0.9	
Mental retardation (F70)	4	2.3	3	2.6	
ASD (F84)	0	0	5	4.3	
ADHD (F90)	0	0	1	0.9	
Epilepsy (G40)	2	1.2	0	0	
History of suicide attempts					
No	53	30.6	36	31.0	*p* = 1.0^a^
Yes	120	69.4	80	69.0	

^a^ χ^2^ test.

^b^ *t*‐test.

**TABLE 3:** Comparisons in benzodiazepine use (dose and polypharmacy) between Periods 1 and 2.

	Period 1 (2013–2015) *N* = 112	Period 2 (2018–2020) *N* = 89	*p* * (corrected)
Mean daily diazepam equivalent dose mg (SD)	32.0 (33.3)	25.6 (30.0)	*p* = 0.0096 (*p* = 0.048)
Mean number of concomitant medications (SD)	2.8 (1.4)	2.0 (1.0)	*p* = 0.0002 (*p* = 0.001)

* Mann–Whitney *U*‐test (Bonferroni correction).

## 4 Discussion

To our knowledge, this is the first study that examined the possible effects of governmental control over benzodiazepine prescription on suicidal behavior in Japan. The annual number of suicides in Japan has risen to approximately 30 000 since 1998. Thereafter, the number of suicides in Japan had been persistently above 30 000, and it began to decrease after 2012 and has been rising again in 2020, probably due to the outbreak of the coronavirus disease 2019 (COVID‐19) pandemic [12, 13]. The number of patients admitted to our ICU who attempted suicide also showed a decreasing trend between Periods 1 and 2, and the percentage of patients who attempted suicide with overdose medication of prescribed drugs decreased significantly from Period 1 to Period 2. Regarding benzodiazepine prescription, both dose and the number of concomitant medications significantly decreased, which is consistent with the results of the previous study [6].

Because of the high suicide rates in Japan, the Basic Law on Suicide Prevention was enacted in 2006, followed by its amendment in 2016. These led to the 2010 alert regarding suicides by overdose medication of psychotropic drugs and the 2012 regulation to control psychotropic drug prescriptions by reducing medical insurance fees and three subsequent reinforcements (2014, 2016, and 2018), which may have contributed to minimizing benzodiazepine prescriptions, thereby reducing psychotropic drug overdoses observed in our study.

It is of note that benzodiazepines increase suicide risk through mechanisms such as promotion of impulsivity and aggression, withdrawal symptoms, and toxicity in overdose [14]. Nevertheless, benzodiazepines are widely used in Japan and may be perceived as relatively safe among psychotropic drugs. As a result, physicians prescribe benzodiazepines in response to patients' complaints of anxiety, insomnia, and autonomic nervous system symptoms, and often find themselves prescribing multiple drugs at large doses [15]. In other words, a substantial proportion of physicians in Japan may not pay much attention to the side effects of benzodiazepines; that is, they may tend to have little regard for not only common side effects such as dependence, abuse, drowsiness, muscle relaxation, and delirium, but also for “paradoxical reaction” or impulsive behaviors (violence, self‐injurious and suicidal behavior) resulting from lack of inhibition [16, 17, 18, 19, 20]. Several risk factors have been cited for paradoxical reactions, including environmental or interpersonal conflicts, inherently hostile or aggressive personality, and poor impulse control [21, 22]. It is conceivable that patients under psychiatric care often have these risk factors, and that benzodiazepines may induce impulsive behaviors due to loss of inhibition. In fact, Gardner et al. [23] reported in a randomized clinical trial in patients with borderline personality disorder that 58% of the alprazolam‐treated patients experienced impulsive behaviors, such as overdose medication, wrist cutting, and violence, which was significantly higher than in the placebo group (13%).

The present study has several limitations. First, we did not take any account of social factors such as economic status in Japan; that is, the unemployment rate is known to be closely related to the annual number of suicide victims [24]. It is possible that differences in unemployment and other social factors between Periods 1 and 2 may have influenced suicidal behavior. Second, the pharmacological effects of concomitant medications such as antidepressants and antipsychotics were not taken into consideration. In addition, this study dealt only with the overdose medication of prescribed psychotropic drugs, but not that of over‐the‐counter (OTC) drugs. It will be of clinical importance to investigate OTC drugs as a possible alternative to benzodiazepines for overdose medication. Third, we compared the period immediately following the prescription restrictions on benzodiazepine drugs (2013–2015) with the period following the tightening of restrictions (2018–2020). To evaluate the impact of the restrictions, it might have been better to compare them with the period immediately prior to the restrictions. Finally, since this study is a single‐center retrospective study, caution is required to generalize the results.

## 5 Conclusions

Our results suggest that suicide attempts by overdose of prescribed psychotropic drugs decreased after the governmental regulation and its reinforcements in Japan. The amount of benzodiazepine prescriptions and the number of concomitant medications also decreased after the regulation in patients with attempted suicide. These results support the possibility that the Japanese policy of reducing benzodiazepine prescriptions was effective in reducing suicide attempts by prescribed psychotropic drugs.

## Author Contributions

A.A. developed the study concept and design, collected and interpreted the data, performed the statistical analyses, and wrote the manuscript. K.M., Y.M., and R.K. collected the data. H.K. interpreted the data, wrote, and revised the manuscript.

## Ethics Statement

The research ethics committee at Teikyo University School of Medicine approved this study protocol (approval number: Teirin No. 21‐716).

## Consent

Informed consent was obtained in the form of an opt‐out option on the Teikyo University ethics committee's website.

## Conflicts of Interest

The authors declare no conflicts of interest.

## Data Availability

According to the study protocol approved by the institutional ethics committee, we did not obtain consent from the subjects as to publication of individual data. Therefore, we cannot disclose the individual data to the public. However, we will respond to data disclosure if there is a request from other researchers.
